# Antenatal Diagnosis of a Rare Neural Tube Defect: Sincipital Encephalocele

**DOI:** 10.1155/2015/613985

**Published:** 2015-07-29

**Authors:** Mehdi Kehila, Sana Ghades, Hassine Saber Abouda, Aida Masmoudi, Mohamed Badis Chanoufi

**Affiliations:** ^1^C Unit, Tunis Maternity and Neonatology Center, Tunis El Manar University, 1029 Tunis, Tunisia; ^2^Foetopathology Department, Tunis Maternity and Neonatology Center, Tunis El Manar University, 1029 Tunis, Tunisia

## Abstract

*Context*. Fetal sincipital encephalocele is one of the most serious congenital neural tube defects with a high risk of mortality and neonatal morbidity. Prenatal diagnosis of this malformation is important in fetal medicine. *Case Report*. We report a case of prenatal diagnosis of sincipital encephalocele using ultrasound and MRI imaging. The diagnosis was done at 25 weeks of gestation by identifying an anterior cephalic protrusion through a defect in the skull. *Conclusion*. Through this case, we discuss the differential diagnosis, management, and prognosis of such lesions.

## 1. Introduction

Encephalocele is characterized by a protrusion of the brain and/or meninges through a defect in the skull that is covered with skin. It is one of the most severe neural tube defects with a prevalence estimated to be 0.8 to 5/10 000 births [1]. Anterior encephaloceles are rare and can be divided into numerous types: sincipital (frontoethmoidal) and basal (transsphenoidal, sphenoethmoidal, transethmoidal, and sphenoorbital). Frontoethmoidal encephalocele (sincipital) is the commonest type of anterior encephaloceles occurring in approximately 1 out of every 5000 to 10 000 live births [1, 2]. This fetal malformation seems to be more common in South East Asia including some parts of India and Thailand [3, 4]. We report a case of anterior encephalocele and discuss the differential diagnosis, management, and prognosis of such lesions.

## 2. Case Report

**Figure 1:**
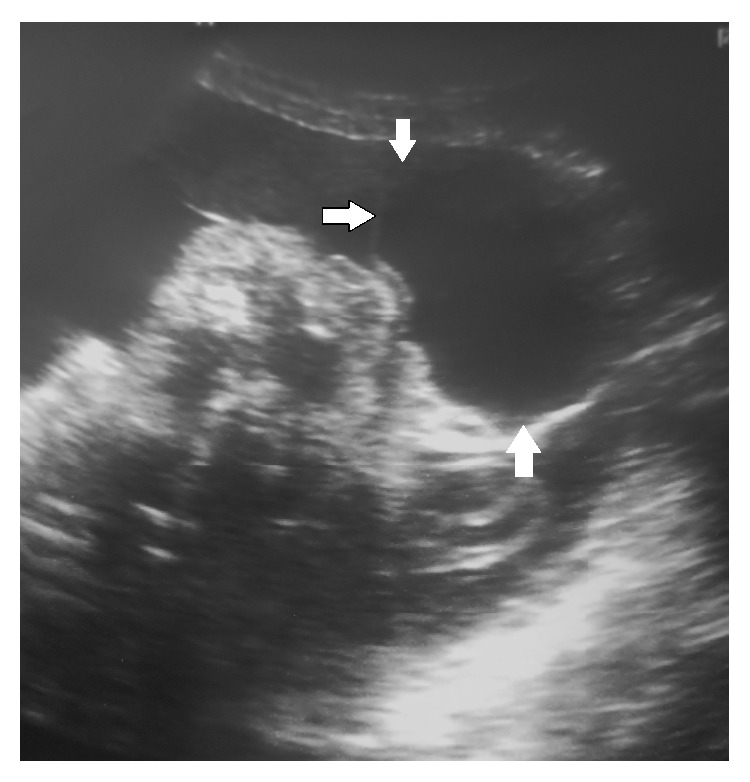
Sonographic examination showing a regularly shaped, midline, facial cystic mass.

**Figure 2:**
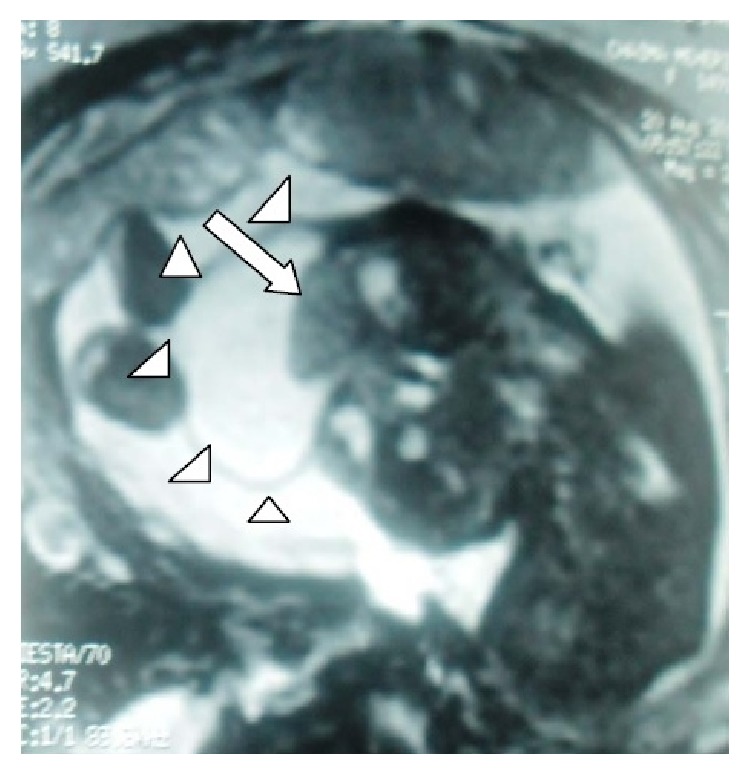
Prenatal MRI showing a small round midline lesion with herniation of brain parenchyma (arrow).

**Figure 3:**
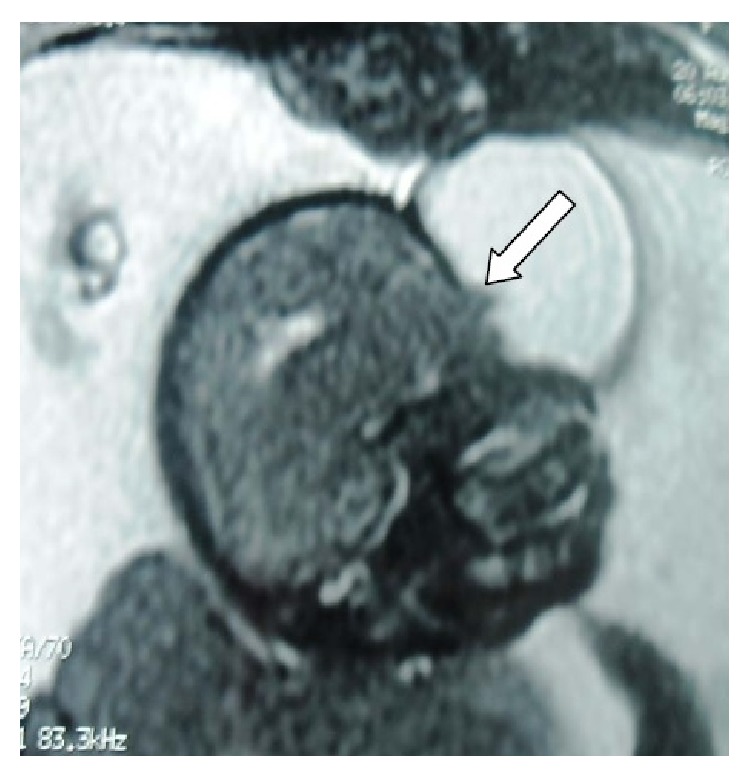
Prenatal MRI showing a well-defined osseous defect in the underlying calvarium (arrow).

A 26-year-old woman, secundigravida, presented for a first medical visit to our department at 22 weeks of gestation for a prenatal exam. She had a sonographic examination four days later. The ultrasound showed a single viable fetus with a regularly shaped, heterogenic, midline, facial cystic mass with no vascular supply (Figure 1). The lesion measured 44 mm in diameter. A brain parenchyma herniation was identified in this mass. The underlying posterior fossa structures including the cerebellar hemispheres and cistern magna did not reveal any definite abnormalities. No fetal extracranial congenital malformations were identified. The diagnosis of a frontal encephalocele was made. An MRI was done for further evaluation of the calvarial defect and the underlying brain parenchyma and showed a small round midline lesion. A well-defined osseous defect was noted in the underlying calvarium. A definite herniation of brain parenchyma was detected in the study (Figures 2 and 3). The fetal brain parenchymal and extraparenchymal structures, including the posterior fossa, ventricular system, and corpus callosum, were within normal limits. There were no signs of neural tube defect. The fetal trunk and extremities were normal. An amniocentesis was performed and revealed a normal male karyotype (46 XY).

Under the diagnosis of fetal isolated encephalocele, counseling by pediatric specialists (neurosurgeons and neonatologists) the mother and the husband followed regarding the prognosis, progress, and postnatal treatment. The parents were informed that even though the survival rate had increased in the anterior defects, the bigger the herniated sac is (in this case 44 mm), the greater the neurological and physical deficits are with or without surgery.

The mother has voiced understanding of the very limited clinical information concerning the long-term effect of such an anomaly on central nervous system development, and she also is aware of the uncertainty of the long-term neurologic outcome with such structural abnormalities and decided for termination of her pregnancy.

**Figure 4:**
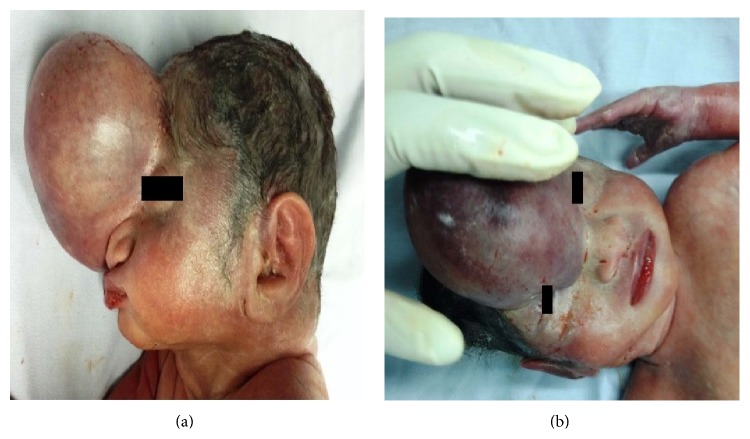
Fetal macroscopic examination showing the sincipital encephalocele.

The assisted abortion was agreed on by the maternity center of ethics committee. The medical termination of her pregnancy by induction of labor was carried out at 25 weeks of gestation. The procedure started by an intracardiac KCl injection followed by misoprostol administration 400 *μ*g by sublingual route every three hours conforming to the FIGO recommendations. The expulsion of the fetus was obtained within 24 hours and the pathological examination of the fetus at autopsy revealed a 650-gram male fetus with a sincipital encephalocele and a frontal bone defect (Figures 4(a) and 4(b)). No other associated abnormalities were detected.

## 3. Discussion

Anterior encephaloceles result from a herniation of intracranial contents through a cranial skull defect with a persistent connection to the subarachnoid space. Based on the location, they can be classified as occipital (75%), sincipital (15%) or basal (10%) [5].

The sincipital meningoencephalocele is typically divided into 3 types [5]:

- Frontoethmoidal (subdivided into nasofrontal (40–60%), nasoethmoidal (30%), and nasoorbital (10%)).
- Interfrontal.
- Those associated with craniofacial clefts.

Frontoethmoidal encephalocele is the most common of the anterior encephaloceles, followed by the nasopharyngeal and orbital types [5–7]. Interfrontal encephalocele is rarer [5].

The Foetopathology Department of Maternity Center of Tunis, Tunisia, drains all of the capital malformed fetuses. Roughly 800 fetopathological exams per year are carried out in this center. Two interfrontal encephalocele cases were detected in 7 years, which estimates the frequency of the disease at 3.6 per 10 000 malformed cases.

Prenatal diagnosis of encephalocele is commonly accomplished by maternal screening of serum *α*-fetoprotein levels and ultrasound (US). Maternal serum and amniotic fluid levels of *α*-fetoprotein are normal when lesions are completely epithelialized [6].

With two-dimensional ultrasound (2D US), encephalocele appears as a defect in the calvarium containing a cystic or solid mass with a gyral pattern that is in continuity with the brain [8]. Prenatal 2D US detects approximately 80% of encephaloceles [9]. The diagnosis is usually easily made from sonographic findings during the second trimester and can also be made in the first trimester. Our patient did not have any prenatal exam in her first trimester. She consulted for the first time at 22 weeks of gestation and the diagnosis was made by US. Prenatal detection of fetal encephalocele has been made by 2D US since 1992 [10], and the first case illustrated by 3D US has been reported in 2006 [11].

Prenatal US, usually, can define the calvarial deficiency, but difficulties might be incurred if the defect is small, fetal head position is poor, or defect is contiguous with nasal cavity [10]. Also, although US can identify presence of brain tissue in the defect, the associated anomalies and exact pathology are not well delineated [10]. The big size of the herniated sac in our case made it easy for the diagnosis to be made, but the MRI scan was better in providing exquisite detail of the cranial defect and the herniated contents.

MRI is increasingly used to evaluate the fetal brain, particularly when an abnormality has been detected on prenatal US or when a fetus is at increased risk for neurodevelopment anomalies. Fetal MRI has several advantages over US. It has higher contrast resolution and is not affected by the shadowing from the calvarium or by low amniotic fluid volume; it allows a larger field of view and can easily be performed using ultrafast T2-W sequences [12]. These advantages make MRI useful in late pregnancy [12].

In terms of the sincipital encephalocele, fetal MRI can provide superior definition of brain and vascular structure images involved and their relative relationship. It can also detect additional central nervous system malformations. Therefore, it can provide important prognostic information for prenatal management. Fetal MRI is also useful in differentiating small cephaloceles from subcutaneous skull cysts and cranial hemangiomas [13, 14]. Because long-term outcome is predicted by severity of microcephaly, amount of herniated tissue, and other intracranial anomalies, use of fetal MRI in association with US is helpful [12, 13].

Encephalocele is frequently associated with other malformations that may be part of recognized syndromes. The most common of the associated syndromes is Meckel-Gruber syndrome which includes occipital encephalocele, microcephaly, microphthalmia, polycystic kidneys, ambiguous genitalia, polydactyly, cleft lip and cleft palate, and other malformations. Other cerebral malformations are often associated with encephalocele such as hydrocephalus, corpus callosal abnormalities, and cerebral dysgenesis [15].

If the size of the sac is important and encephalocele is bulky, with severe microcephaly or other lethal anomalies, termination of pregnancy may be the choice due to the severe morbidity and mortality [16]. However, postpartum surgical treatment is appropriate for cases with relatively small encephalocele and without other associated lethal anomalies. The procedure basically consists of removing the overlying sac and closing the defect including the dural defect [17]. Therefore, vaginal delivery may be considered if the lesion is relatively small [17].

Specific information on the outcome of a child with a cephalocele can be difficult to find and interpret. French reported that 83% of patients with encephaloceles had mental handicap and/or physical impairment [18]. Brown and Sheridan-Pereira [19] found, in a series of 34 infants with cephalocele, 29% mortality in children with posterior cephaloceles compared with 0% mortality associated with anterior defects. In a retrospective cohort study, about 85 cephalocele cases treated at the Hospital for Sick Children (Toronto, Canada) between 1990 and 2006, cognitive development was abnormal in 52% of patients with mild, moderate, or severe mental retardation in, respectively, 11%, 16%, and 25% of patients [16].

## 4. Conclusion

Sincipital encephalocele is rare and should be suspected on the basis of any anterior midline mass. The diagnosis can be made by US. Fetal magnetic resonance imaging (MRI) may provide superior detail of central nervous system (CNS) anomalies. The extent of cerebral tissue involvement in an encephalocele is also better defined with MR imaging, which aids in prognosis and surgical planning. The prognosis of babies with encephalocele is variable. Babies with an encephalocele at the back of the head have a 55 percent survival rate. Long-term prognosis for survival becomes less likely if there are other complications. Approximately 75 percent of these infants who do survive have varying degrees of mental deficit.
